# Agricultural Pesticide Use in Malawi

**DOI:** 10.5696/2156-9614-8.20.181201

**Published:** 2018-12-03

**Authors:** Jacob Jeketule Soko

**Affiliations:** Jomo Kenyatta University of Agriculture and Technology, Nairobi, Kenya

**Keywords:** Pesticide use, pesticides, health risk, integrated pest management, health, systematic literature review, Malawi

## Abstract

**Background.:**

The majority of farmers in Malawi use pesticides to protect their crops from pests. Without the use of pesticides, farmers would not be able to harvest significant yields. However, several studies worldwide have shown that some pesticides used by farmers are harmful to human health. Despite these studies, there remains a lack of proper documentation of the use and nature of harmful pesticides.

**Objectives.:**

This retrospective study aimed to explore agricultural pesticides used in Malawi, to investigate factors that make Malawi vulnerable to illegal pesticide use, and to assess the extent that farmers in Malawi have adopted integrated pest management (IPM).

**Methods.:**

We reviewed the literature and empirical studies relating to the effects of pesticides on human health. Three databases were searched: EBSCOhost, JSTOR and Africa Journals Online (AJOL). Secondary data were used in the present study, such as case studies, reports and published research studies prior to 2010. We used three search terms: “pesticides causing death in Malawi”, “effect of integrated pest management plan”, and “pesticides that cause harm to humans in Malawi”.

**Discussion.:**

The studies revealed that farmers in Malawi use insecticides, fungicides, herbicides, fumigants, nematicides, acaricide, and rodenticides. These chemicals are mainly used on tobacco, tea, sugarcane, coffee, cotton, and maize crops. Furthermore, the study revealed that farmers in Malawi obtain illegal pesticides from vendors from neighboring countries and that the integrated pest management plan has not been successful in Malawi.

**Conclusions.:**

The present study recommends that the agricultural department should devise strategies to increase understanding of the effects of pesticides, restrict illegal pesticides and implement procedures curbing illegal pesticides and policies to support integrated pest management.

**Competing Interests.:**

The authors report no competing financial interests.

## Introduction

**Figure 1:**
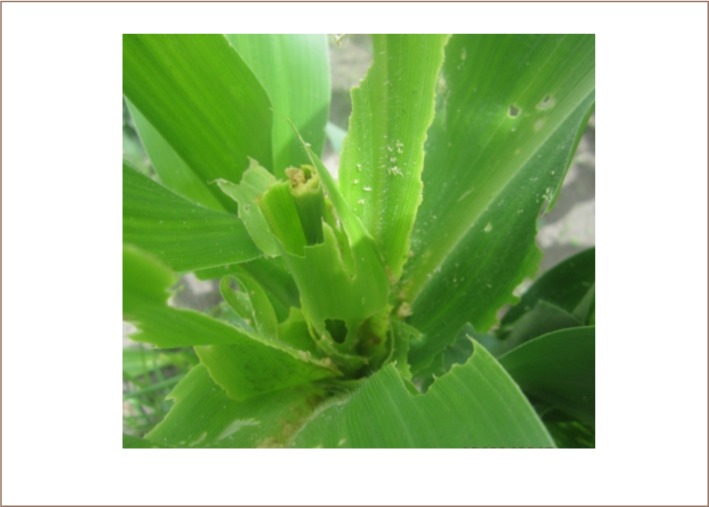
Maize plant infected by armyworms

Pesticides are substances used in agriculture to increase crop yields and improve the appearance of plant products, as well as other uses.^1^ According to Langley and Mort, pesticides are used in most homes, businesses and farms to control pests, including insects, weeds, fungi, rodents, and even microbial organisms.^2^ According to Lakudzala, rapid agricultural development in Malawi has led to an increased use of pesticides.^3^ At least 2000 metric tons of pesticides are used annually, 70% of which are used for agriculture (*Figure 1*).^3^

While pesticides are used to destroy pests, reports show that some of the pesticides are harmful to human health and the environment. Several studies support the assertion that pesticides are the most common poisons used throughout the tropics and are associated with high mortality rates.^1^,^4^,^5^ Furthermore, a number of studies have established the direct effect of pesticides on health, but only a few studies have reported on the indirect relationship between pesticides and health connecting pesticide use to sicknesses. However, as noted by the United Nations Special Rapporteur, it is difficult to obtain reliable, global statistics on the number of people who suffer from pesticide exposure, hence studies on pesticides statistics rely mostly on estimates.^6^

Although the majority of pesticides (80%) are used in high-income countries, most cases of poisoning occur in low-income countries.^7^ According to a report by the United Nations, approximately 200,000 people die worldwide annually from toxic exposure to pesticides.^6^ Gunnell et al. reported that most of the cases of pesticide self-poisoning occur in agricultural communities in low- and middle-income countries.^4^ According to Binns, Dixon and Nel, over 11 million cases of poisoning in Africa each year can be attributed to exposure to harmful pesticides.^8^ In sub-Saharan Africa, pesticides are being used more frequently by small farmers in an unsustainable way.^9^ Approximately 62% of Malawi's population depends on agriculture as a means of livelihood, and pesticide use is common.^10^ The major crops grown in Malawi include tobacco, sugarcane, coffee, maize, beans, groundnuts, cotton, and tea.

### Unintentional-indirect poisoning

Pesticides constitute a health risk to humans, domestic animals, wildlife and other non-target organisms in the environment.^2^ Abhilash and Singh observed that exposure to pesticides is increasingly linked to immune suppression, hormone disruption, diminished intelligence, reproductive abnormalities and cancer.^11^ Similarly, Stadlinger et al. stated that there is increasing evidence suggesting that pesticides have intrinsic public health and environmental risks during their production, import, use, storage and disposal.^12^

### Accidental poisoning

Several studies indicate that pesticides have led to acute poisoning, which can be categorized as accidental or suicidal cases.^4^,^13^,^14^ A study by Gunnell et al. found out that pesticide self-poisoning accounts for about one-third of the world's suicides and epidemiological and toxicological data suggest that many of these deaths might be prevented if the use of pesticides most toxic to humans was restricted, pesticides could be safely stored in rural communities and accessibility and quality of care for poisoning could be improved.^4^ In Malawi, it is estimated that pesticide self-poisoning constitutes around 80% of suicides.^15^

In order to mitigate the harmful effects of pesticides, some countries have implemented the practice of integrated pest management (IPM)^16^ Integrated pest management has the potential to reduce deaths that result from pesticide and chemical use. According to Pretty and Bharucha, IPM is the deployment of a variety of methods of pest control designed to complement, reduce or replace the application of synthetic pesticides.^16^ Integrated pest management includes structural and procedural improvements to reduce food, water, shelter and access to pests.^17^,^18^

**Abbreviations**
*AJOL*: Africa Journals Online
*DDT*: Dichlorodiphenyltrichloroethane
*IPM*: Integrated pest management
*PRISMA*: Preferred Reporting Items for Systematic Reviews and Meta-Analyses

Given the numerous reports and cases of pesticide exposure, Malawi faces the challenge of selecting low-risk pesticides. This study is a retrospective approach that seeks to explore agricultural pesticides used in Malawi, investigate factors that make Malawi vulnerable to illegal pesticide use, and to assess the extent to which farmers in Malawi have adopted IPM measures and their success.

## Methods

The present study involved a systematic review of the published literature on empirical studies relating to the effects of pesticides on human health. The objective of the present study was to address three questions: (a) What pesticides being used in Malawi have potential health risks? (b) What factors make Malawi farmers vulnerable to pesticides with potential health risks? and (c) To what extent have farmers in Malawi adopted integrated pest management plans?

We used the Preferred Reporting Items for Systematic Reviews and Meta-analyses (PRISMA) guidelines. Systematic reviews attempt to gather all relevant information that fits pre-specified eligibility criteria to answer a specific question. In addition, systematic reviews use explicit systematic methods to minimize bias in the identification, selection, synthesis and summary of studies. Meta-analysis is the use of statistical techniques to combine and summarize the results of multiple studies and provide more precise estimates of the effects on health than those derived from individual studies. PRISMA is recommended by Moher for organizing systematic reviews.^19^

We searched three databases: EBSCOhost, JSTOR and Africa Journals Online (AJOL). EBSCOhost was chosen because it is the leading research database for general and specific medical institutions related to the present study. JSTOR was chosen because of its current journal issues. Finally, AJOL was selected because its journals provide an African context. AJOL was determined to be an appropriate database for this study, especially in terms of pesticides in Malawi. Secondary data were used in the present study as well, such as case studies, reports and published research articles prior to 2010.

**Figure 2:**
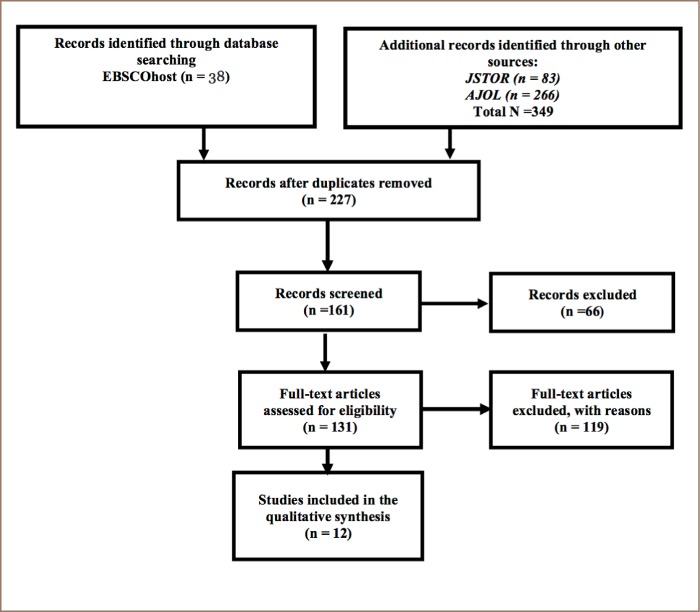
Flowchart showing literature search process

Three search terms were used: “pesticides causing death in Malawi”, “effect of integrated pest management plan” and “pesticides that cause harm to humans in Malawi”. Furthermore, the expression “pesticides AND Malawi” was also used in the database searches. Figure 2 shows literature search process using EBSCOhost, JSTOR and African Journals Online search engines.

## Results

In this systematic review, the EBSCOhost database search yielded 38 results, and JSTOR and AJOL produced 83 and 266 results, respectively. After removing duplicate records, there were 227 records. A total of 66 records were excluded because they did not contain full texts, leaving 131 full-text articles that were assessed for eligibility. Of these studies, 119 articles were excluded because they did not relate directly to the research questions, leaving 12 studies that were included in the analysis.

### Pesticide use in Malawi

To determine the pesticides used in Malawi, articles that matched the criteria for selection were reviewed systematically (*Table 1*). Farmers in Malawi practice both subsistence and commercial farming, which use large amounts of pesticides. We determined that insecticides are used most often in Malawi, followed by herbicides, fumigants, fungicides and rodenticides. To our surprise, we further determined that in some parts of Malawi, people consumed pesticide-treated seedlings, fully knowing that these seedlings could affect their health.

**Table 1:**
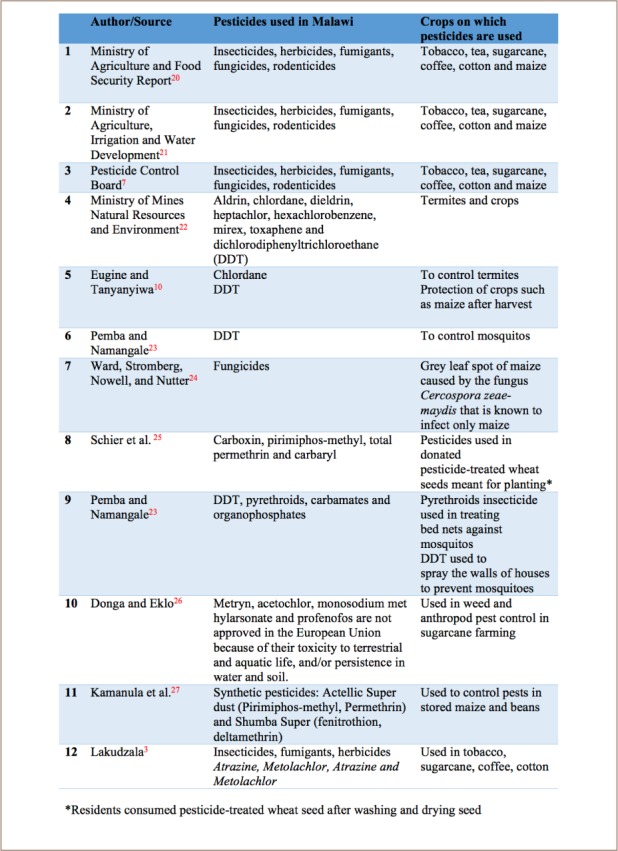
Literature Survey of Pesticides Used in Malawi

We further established that herbicides are primarily used on sugar plantations, whereas fumigants are primarily used in the tobacco industry. We observed that insecticides are primarily used for field crops, specifically maize. Furthermore, according to the Pesticide Control Board, pesticides were mostly used in tobacco, followed by tea and sugarcane and least used in maize, followed by cotton and coffee.^7^

### Factors contributing to illegal pesticide use

To determine the factors that contribute to Malawi being vulnerable to illegal pesticides, articles that matched the criteria for selection were systematically reviewed. We determined that several factors could be summarized into three themes. First, there is the illegal importation of pesticides as Malawi does not manufacture its own pesticides and hence relies on imported pesticides. This situation makes it difficult for Malawi to regulate pesticides. In addition to that, Malawi does not have ultimate pesticides disposal facilities (such as pesticide incinerators).^21^

### Integrated pest management plan

To determine the extent to which farmers in Malawi have adopted an integrated pest management plan, several articles that matched the criteria for selection were systematically reviewed. Few documents included an assessment of IPM. A majority of the documents discussed the nature and implementation of IPM plans. Among the studies that assessed IPM, a study by Pretty and Bharucha assessed the successes of IPM in four types of IPM projects and determined that at least 50% of the pesticides used are not needed in most agro-ecosystems.^16^ The study further revealed that policy support for IPM is relatively rare. The study also exposed counter-interventions from the pesticide industry. Finally, the biggest challenge identified for IPM was that pests, diseases, and weeds evolve and move.^16^

## Discussion

In this systematic review examining pesticides that are harmful to human health in Malawi, 12 studies were reviewed. The reviewed literature highlighted important issues for policymakers: (a) Malawi uses a variety of pesticides that are prone to causing harm, (b) Malawi still uses some banned pesticides, (c) Malawi is vulnerable to infiltration of pesticides from neighboring countries, and (d) IPM plans have not been successful.

### Pesticide use in Malawi

First, farmers in Malawi depend entirely on a variety of pesticides, namely, insecticides, without which sufficient yields would not be realized. Several studies state similar findings, for example, a study by Action on Smoking and Health (2017) noted that most pesticides were used for tobacco, which is a sensitive plant to grow, and requires multiple pesticides, fungicides, and herbicides throughout its growing season. A report by the World Health Organization pointed out that tobacco is often grown without rotation with other crops (i.e. as a monocrop), leaving the tobacco plants and soil vulnerable to a variety of pests and diseases and dependent on pesticides for survival.^28^ Many of these chemicals are so harmful to both the environment and farmers' health that they are banned in some countries. In low- and middle-income countries, pesticides and growth inhibitors are usually applied with handheld or backpack sprayers, without the use of the necessary protective equipment, making skin and respiratory exposure to the toxic chemicals more likely.

The present study also determined that farmers in Malawi still use some of the chemicals banned in 1984 and 1985.^22^ These pesticides include chemicals such as aldrin, chlordane, dieldrin, heptachlor, hexachlorobenzene, mirex, toxaphene and dichlorodiphenyltrichloroethane (DDT). These chemicals were found to be persistent, and safer alternatives are preferred.^22^ Although some persistent organic pollutants (POPs) were banned from being used in the country, illegal use and trading of POPs are prevalent in Malawi.

A study by Eugine and Tanyanyiwa revealed the presence of chlordane, which is a common pesticide used to control termites mainly used by the construction industry.^10^ The authors note that chlordane is primarily sold by market vendors, hardware shops, and agro-dealers. The survey also revealed that the reappearance of DDT is due to products coming across the border with Mozambique. DDT is used by individuals and farmers to control termites and protect crops such as maize after harvest. Studies on the power utility revealed that between 2003 and 2005, approximately 395,000 liters of polychlorinated biphenyls (PCBs)-containing oil were received in Malawi and traces of PCBs remain in many sites in Blantyre, Malawi.

Fungicides have been found to provide excellent control of grey leaf spot, which is considered one of the principal constraints to maize production in sub-Saharan Africa. Grey leaf spot is caused by the fungus Cercospora zeae-maydis, which is known to infect only maize. Following periods of high humidity in Malawi, maize yield incurs losses of 29–69% due to grey leaf spot.^24^

### Factors contributing to illegal pesticide use in Malawi

Malawi does not manufacture pesticides; hence, it relies on imported products.^20^ In addition to being a landlocked country, Malawi faces the challenges of unregulated and illegal chemical pesticides which enter the country through its borders with Tanzania, Mozambique, and Zambia. For example, DDT was identified as reappearing in the country with its source linked to Mozambican traders.^10^,^22^ Furthermore, the Ministry of Mines Natural Resources and Environment acknowledges that there is always a high demand for pesticides, and as a result, there is a substantial illegal trade of pesticides.^22^ Although the Ministry of Agriculture in Malawi is trying to control the use of pesticides through the Ministry of Agriculture, Irrigation and Water Development, illegal pesticide use remains and the situation is worsening. Some of these illegal pesticides are dangerous and instead of making large profits from crops, farmers have reported painful losses caused by the chemicals. Another report states that the illegal trade in pesticides is a significant global problem. In addition, a report from Malawi showed that some farmers in Malawi use expired chemical pesticides.^29^ These studies concur with the findings of Vaagt who noted that in developing countries, as much as 30% of pesticides do not meet internationally recognized safety standards.^30^

### Integrated pest management plan

We were not able to determine the extent to which farmers in Malawi have adopted an integrated pest management plan. Similarly, Pretty and Bharucha observe that evidence for IPM's impacts on pesticide use and yields remains patchy.^16^

### Quality and strength of body of evidence

As observed in Supplemental Material, only 11 sources were found to be relevant to objective one, examination of pesticide use in Malawi. Three sources explored factors that make Malawians vulnerable to illegal pesticides, four sources related to the extent Malawi farmers have adopted integrated pest management, and two sources were connected to emerging themes. Some literature did not relate directly to the study objectives but strengthened the arguments on magnitude of the problem of pesticides. Furthermore, three of the sources were comprised primarily of government documents from the Ministry of Agriculture and Food Security, Ministry of Agriculture-Irrigation and Water Development, and Ministry of Mines Natural Resources and Environment. This means that there are no wide-ranging sources on pesticides in Malawi available in the literature. However, all of the available documents were in general agreement on pesticide use in Malawi.

As for objective two, factors that make Malawi vulnerable to illegal pesticides, three relevant sources were identified. All three were from government documents. In relationship to objective three, four relevant sources were found. Two of these sources were government documents, while the other two came from other sources. Finally, relating to the emerging theme of illegal pesticides, two sources were identified and both were government documents. In addition, a number of sources explored the concept of IPM and its implementation, but few studies evaluated its effectiveness. This could be because the IPM was only implemented in 2015. Overall, there is little documentation about pesticides used in Malawi and IPM.

## Conclusions

The present study was a systematic review of the literature and empirical studies relating to the effects of pesticides on human health. We concluded that farmers in Malawi use insecticides, fungicides, herbicides, fumigants, nematicides, acaricide, and rodenticides, and these pesticides are mainly used for tobacco, tea, sugarcane, coffee, cotton, and maize crops. Additionally, the study concluded that farmers in Malawi obtain illegal pesticides from vendors from neighboring countries, and that an integrated pest management plan has not been successful in Malawi. Based on these findings, we recommend the following: (a) increasing the understanding of the effects of pesticides in order to minimize their usage, (b) restricting illegal pesticides and implementing policies to this end, and (c) pursuing policies to support integrated pest management. Regarding the quality of body of evidence, there is little literature concerning pesticides in Malawi. The majority of sources were from government documents, making it difficult to verify the results with a wide-ranging body of literature.
