# Supplementary material for: Agricultural Pesticide Use in Malawi
Source: J Health Pollut. 2018 Dec 3;8(20):181201. doi: 10.5696/2156-9614-8.20.181201 (PMC6285677; doi:10.5696/2156-9614-8.20.181201)
Supplement: Supplementary file 1 [file hapn-8-20-181201_s01.docx]

**Supplemental Material**

**Agricultural Pesticide Use in Malawi**

Jacob Jeketule Soko

**Quality and Strength of Body of Evidence**

| # | Author | Sources related to objective one: Examination of pesticides used in Malawi | Sources related to objective two: Exploration of factors that make Malawians vulnerable to illegal pesticide use | Sources related to objective three: Extent that Malawi farmers have adopted integrated pest management | Sources relating to emerging theme –illegal pesticides | Literature not directly related to study objectives |
| --- | --- | --- | --- | --- | --- | --- |
| 1 | Abhilash and Singh^11^ |  |  |  |  | Effects of pesticides |
| 2 | Bertolote et al.^14^ |  |  |  |  | Accidental poisoning |
| 3 | Binns et al.^8^ |  |  |  |  | Statistics on poisoning cases |
| 4 | Cha et al.^5^ |  |  |  |  | Magnitude of pesticide usage |
| 5 | de Bon et al.^9^ |  |  |  |  | Pesticide use by small-holder farmers |
| 6 | Donga et al.^26^ | Yes |  |  |  | Pesticide use by small-holder farmers |
| 7 | Dzamalala et al.^15^ |  |  |  |  | Pesticides and self-poisoning |
| 8 | Eugine and Tanyanyiwa ^10^ | Yes |  |  |  |  |
| 9 | Gunnell et al.^4^ |  |  |  |  | Statistics on accidental poisoning |
| 10 | Kamanula et al.^27^ | Yes |  |  |  |  |
| 11 | Lakudzala^3^ | Yes |  |  |  |  |
| 12 | Langley et al.^2^ |  |  |  |  | Magnitude of pesticide usage in homes |
| 13 | Lekei et al.^13^ |  |  |  |  | Pesticides and poisoning |
| 14 | Ministry of Agriculture and Food Security^20^ | Yes | Yes | Yes | Yes |  |
| 15 | Ministry of Agriculture, Irrigation and Water Development^21^ | Yes | Yes | Yes | Yes | Challenges in pesticide disposal |
| 16 | Ministry of Mines Natural Resources and Environment^22^ | Yes | Yes |  |  |  |
| 17 | Orr^31^ |  |  | Yes |  |  |
| 18 | Pemba et al.^23^ | Yes |  |  |  |  |
| 19 | Dylo et al.^32^ | Yes |  |  |  |  |
| 20 | Pesticides^7^ | Yes |  |  |  | Statistics on usage and pesticide poisoning |
| 21 | Pestizid Aktions-Netzwerk^1^ |  |  |  |  | Advantages of pesticides |
| 22 | Pretty et al.^16^ |  |  | Yes |  |  |
| 23 | SP-IPM^18^ |  |  |  |  | Role of IPM |
| 24 | Stadlinger et al.^12^ |  |  |  |  | Effects of pesticides |
| 25 | Stock^17^ |  |  |  |  | Concept of IPM |
| 26 | United Nations Special Rapporteur^6^ |  |  |  |  | Reliability of global statistics on pesticides |
| 27 | Ward et al.^24^ | Yes |  |  |  | Effects of pests on crops |
| 28 | World Health Organization^28^ |  |  |  |  | Predominant crops on which pesticides are applied |
